# PROMPT: a protein mapping and comparison tool

**DOI:** 10.1186/1471-2105-7-331

**Published:** 2006-07-04

**Authors:** Thorsten Schmidt, Dmitrij Frishman

**Affiliations:** 1 Department of Genome Oriented Bioinformatics, Technische Universität München, Wissenschaftszentrum Weihenstephan, 85350 Freising, Germany

## Abstract

**Background:**

Comparison of large protein datasets has become a standard task in bioinformatics. Typically researchers wish to know whether one group of proteins is significantly enriched in certain annotation attributes or sequence properties compared to another group, and whether this enrichment is statistically significant. In order to conduct such comparisons it is often required to integrate molecular sequence data and experimental information from disparate incompatible sources. While many specialized programs exist for comparisons of this kind in individual problem domains, such as expression data analysis, no generic software solution capable of addressing a wide spectrum of routine tasks in comparative proteomics is currently available.

**Results:**

PROMPT is a comprehensive bioinformatics software environment which enables the user to compare arbitrary protein sequence sets, revealing statistically significant differences in their annotation features. It allows automatic retrieval and integration of data from a multitude of molecular biological databases as well as from a custom XML format. Similarity-based mapping of sequence IDs makes it possible to link experimental information obtained from different sources despite discrepancies in gene identifiers and minor sequence variation. PROMPT provides a full set of statistical procedures to address the following four use cases: i) comparison of the frequencies of categorical annotations between two sets, ii) enrichment of nominal features in one set with respect to another one, iii) comparison of numeric distributions, and iv) correlation of numeric variables. Analysis results can be visualized in the form of plots and spreadsheets and exported in various formats, including Microsoft Excel.

**Conclusion:**

PROMPT is a versatile, platform-independent, easily expandable, stand-alone application designed to be a practical workhorse in analysing and mining protein sequences and associated annotation. The availability of the Java Application Programming Interface and scripting capabilities on one hand, and the intuitive Graphical User Interface with context-sensitive help system on the other, make it equally accessible to professional bioinformaticians and biologically-oriented users. PROMPT is freely available for academic users from .

## Background

Molecular bioinformatics was born as a science of comparing individual DNA and amino acid sequences with each other. Over the past three decades important biological insights have been obtained by establishing unexpected sequence similarity between seemingly unrelated proteins (e.g., Koonin *et al. *[1]). More recently, modern high-throughput technologies (genome sequencing, expression profiling, mass spectrometry) injected tremendous amounts of sequence data and associated experimental information into the public databases, creating the need for collective comparisons of large sequence groups (e.g., whole proteomes). The transition from pairwise sequence comparison to comparing large protein datasets against each other is similar to switching from finding differences between individuals to comparing populations of whole countries. Is wine consumption in France higher than in England? Do Germans drive faster than Americans? Analogous queries applied to biological molecules prevail in post-genomic bioinformatics. In many genome sequencing papers one finds a bar chart contrasting the new sequence with other genomes in terms of sequence motif composition. While analysing gene clusters obtained by expression analysis it is typical to ask whether one gene group is significantly enriched in certain functional categories with respect to another one. Are proteins with many interaction partners different from less prolific interactors [2]? Are essential genes more evolutionary conserved than non-essential ones [3]? The list of such questions is endless. Answering some of them involves a mere counting exercise while others require the application of sophisticated bioinformatics approaches and careful statistical analyses.

Mining protein properties at large scale has been especially productive in computational structural genomics where it helped to establish basic facts about structural complements encoded in complete genomes. For example, it was shown that membrane proteins constitute roughly 30% of each proteome [4]. The patterns of globular fold occurrence in different organism groups were carefully investigated [5]. The mechanisms of protein structure adaptation to extreme environments were revealed by comparing the genomes of thermophilic [6,7], halophilic [8], psychrophilic [9], and barophilic [10] species with their counterparts living under normal conditions.

A recurrent bioinformatics task in comparative proteomics involves mapping and integrating information from disparate sources. While reporting experimental results as well as theoretical predictions one may refer to proteins using the UniProt [11], GenBank [12], or RefSeq [13] nomenclature, or custom IDs for sequences not yet submitted to public databases. The situation is additionally complicated by frequent genome updates which may result in new, previously missed ORFs identified, existing sequences corrected, as well as the removal of misannotated ORFs. As a result, establishing unambiguous correspondence between protein sequence entries and associated experimental data may represent a difficult, albeit trivial challenge.

Countless customized software tools with varying degrees of complexity have been independently written in research labs throughout the world to address protein comparison and mapping tasks, although there are significant commonalities in the technical steps that need to be implemented. The authors of this contribution, too, wrote their share of throw-away perl scripts and quick-shot *Java *programs to compare GroEL substrates with the rest of the *Escherichia coli *lysate [14], crystallizable and non-crystallizable proteins [15], disease-associated proteins and those without such association [16], abundant and non-abundant proteins (Ishimama *et al*., in preparation), as well as completely sequenced genomes [17] and functional properties of alternatively spliced genes [18]. It is precisely the fatigue from re-inventing the wheel over and over again that motivated us to develop a bioinformatics framework for large-scale protein comparisons.

Much to our surprise, we realized that general solutions for comparing and analysing large sets of proteins in the space of arbitrary annotation attributes are currently hardly available or limited to certain application areas. We are aware of only two software projects addressing the need for large scale comparative analysis. The comprehensive Genome Properties resource [19] allows comparing complete prokaryotic genomes based on a multitude of pre-defined property assertions. The system is primarily focused on metabolic information, does not allow user-supplied protein attributes, does not provide statistical tests to validate differences between genomes, and is not available for local installation. GeneMerge [20] is an excellent tool for detecting over-representation of certain functional or categorical descriptors in a given subset of proteins relative to the general set based on rigorous statistical tests, but it provides neither integration with bioinformatics databases nor a graphical user interface.

Here we describe a platform-independent system named PROMPT (Protein Mapping and Comparison Tool) capable of addressing a wide spectrum of routine tasks in comparative proteomics. PROMPT enables the user to compare arbitrary protein sequence sets, revealing statistically significant differences in their annotation features. Protein annotation can be imported from a variety of standard bioinformatics databases as well as from generic XML description files. Facilities are provided for linking experimental information obtained from different sources to appropriate genes despite discrepancies in gene identifiers and minor sequence variation. The entire functionality of the system is available via a full-featured server-independent graphical user interface. At the same time, a Java API is provided for integration with user applications.

## Implementation

### Functional overview

PROMPT operates with three types of information associated with proteins: database IDs, amino acid sequences, and annotation attributes. The latter may be any protein feature manually assigned, experimentally measured, or calculated from sequence; such features may be nominal and/or numeric. Examples of numeric features are molecular weight, pI, abundance, and the number of interaction partners. Nominal features can be sequence motifs, keywords, functional categories, EC numbers, and so on. Sequences are primarily used by PROMPT to establish the correspondence between proteins imported from different sources and thus having incompatible database IDs. This is done by similarity-based mapping and careful handling of exceptions and minor sequence variations. Sequence data can be either obtained directly from public databases, or supplied by the user as flat files using one of the commonly accepted formats as well as a custom XML format.

Once annotation features have been imported and assigned to appropriate proteins, actual large scale comparisons of protein properties, data interpretation, and statistical analyses can be conducted. The central task consists of comparing two sets of proteins and finding significantly enriched or depleted features in one of the sets. Results can be viewed in tabular form, visualized by various types of plots, and exported to other applications.

**Figure 1:**
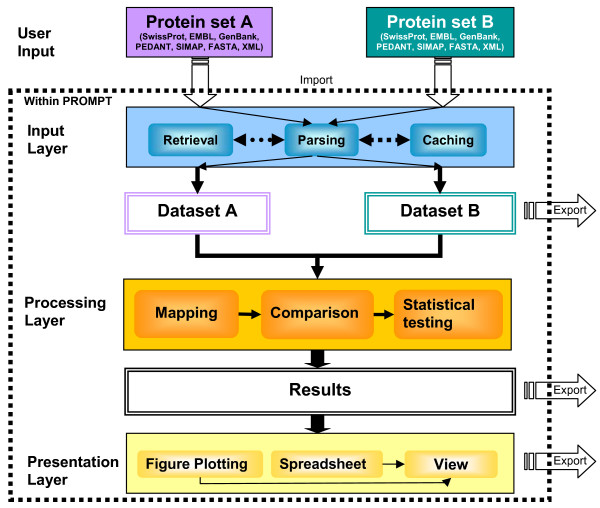
General Workflow of PROMPT.

As seen in Figure 1, a general PROMPT workflow involves three stages: i) data import, ii) data processing which includes mapping, comparison, and statistical tests, and iii) visualization and presentation of results for subsequent analyses. Additionally, the data can be exported and saved at each step.

### Technology

PROMPT is written in Java 1.5. The Graphical User Interface (GUI) was built with Java Swing, and the help system utilizes Java Help Extensions. The Apache log4j package [21] handles message logging and reporting. All input, test, engine and visualisation classes are loaded dynamically by the GUI using Java reflections. Scripting functionality is realized with the BeanShell package [22].

### Software architecture

PROMPT is partitioned into three self-contained layers – the input layer, the processing layer, and the visualization layer- which are interconnected via clearly defined interfaces. These interfaces ensure interoperability between a wide variety of input sources, algorithms, visualisation techniques and export methods by defining cross-layer communication in such a way that an algorithm, once developed, will work with any input module that provides the requested input interface. It does not matter, for example, whether the sequence data comes from a local UniProt XML file [11], an SQL database or a Web service. This approach allows the application of PROMPT's algorithms to new and currently unknown data formats and sources. Conversely, newly added algorithms can immediately reuse all of the available input and output modules. The same applies to new import modules that can be used with all applicable algorithms as soon as the required interfaces have been implemented. Similar to the approach adopted in *Java Beans *[23] all PROMPT modules are encapsulated by the troika of *Init*, *Run*, and *GetResults *methods that perform initialization, actual computation and the returning of results, respectively. This design pattern provides a comfortable and uniform handling of all parts of the PROMPT framework. Furthermore, the clear separation between individual layers ensures reproducibility of results as the data can be saved and evaluated at every step.

### Data retrieval and integration

Data import from flat files is predominantly based on BioJava [24] which is used to parse multi-FASTA, EMBL [25], Genbank [12], and UniProt [11] formats. In particular, the UniProt XML format is supported. Additionally, data can be directly imported from two MIPS databases – PEDANT [17] and SIMAP [26] – using data access objects provided by these two resources. User extensions can be easily incorporated by creating Java classes that implement or extend the Java interfaces provided by PROMPT.

**Figure 2:**
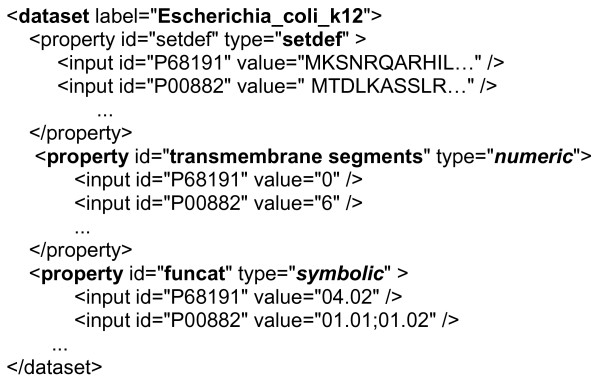
**Example PROMPT XML File**. The file contains a set definition property that encompasses all *E.coli *proteins together with their amino acid sequences. Additionally, annotation information stored in the numeric property *transmembrane segments *and in the symbolic property *funcat *is provided.

Alternatively user-specific data can be loaded in PROMPT's custom XML format. Such an XML file (Figure 2) can contain any number of numeric or nominal attributes for a set of elements that we, for simplicity, assume here to be proteins (but could also be any other kind of object including protein sequence domains, DNA sequences, molecular structures, phenotype data, and so on). A numerical attribute could be e.g. the number of predicted transmembrane segments or molecular weight. Examples of nominal attributes are EC numbers or functional categories. Annotation properties are represented as XML nodes with the name *property*. They have an *id *attribute that serves as a unique reference to the property within the XML file. Additionally, the property nodes have an attribute of the name *type *that can have either the value *numeric *or *symbolic *for numeric or nominal data, respectively. Within the property elements the annotation data for each protein are stored as XML nodes in the form *<input id = "XX" value = "YY"> *where *YY *represents annotation data for the protein with the identifier *XX*. A numerical attribute can be any number in Anglo-Saxon notation, e.g. 10, 0.7, or 1E-6. Nominal attributes of a protein contain one or many arbitrary strings separated by semicolons, e.g. *"energy; metabolism; ATP*". Optionally, XML files can contain a property element of the type *setdef *which defines a set of elements (proteins). A formal Document Type Definition (DTD) of the XML structure is given in the supplementary information [see Additional file 1].

Due to the generic XML import capability the system can be fed with arbitrary annotation without considering its semantics, making PROMPT applicable to data analysis in any knowledge domain, not necessarily limited to molecular bioinformatics. Additionally, data in widely used tab-delimited text and WEKA's ARFF [27] files can be processed. A full list of available data import options can be found in Table 1.

**Figure 3:**
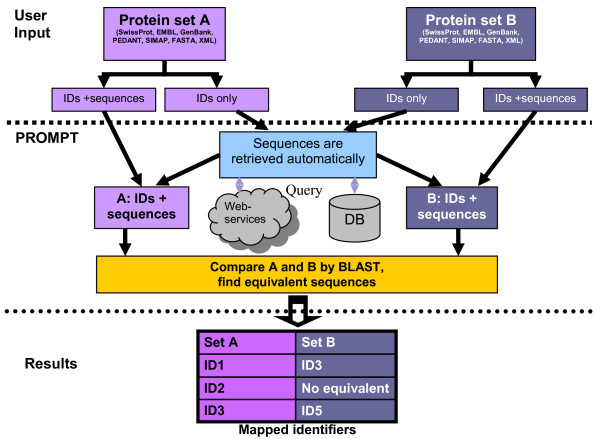
Data input and mapping workflow.

Sequences and annotation available in major public databases may be fetched by their identifiers via the SeqHound [28] web services (Figure 3). All the user needs to do is to supply a list of UniProt [11] or GenBank [12] identifiers and the corresponding information will be downloaded automatically in the background. All actions are tracked by a fully-configurable logging facility; if ambiguous IDs or errors are encountered, warnings will be issued. Remotely retrieved data are cached locally to avoid repeated re-fetching of the same data items during processing.

### Similarity-based sequence mapping

If input data contain proteins with incompatible database IDs, correspondence between individual entries can be established by sequence comparisons. PROMPT automates all-against-all BLAST [29] searches (Figure 3), producing (n*(n-1))/2 alignments, where *n *is the number of proteins in the dataset. The user is then prompted to choose the extent to which sequence differences can be tolerated for specific purposes. The list of typical minor variations between essentially the same gene products includes missing start methionines, different versions of the same genomic ORF, and splice isoforms. For example, the brain tumor protein BRAT_DROME in *Drosophila melanogaster *has seven synonymous UniProt [11] accession numbers and 9 associated GenBank [12] entries; according to UniProt [11] its amino acid sequence has been revised after the primary submission. Using the mechanism described above, a given list of GenBank [12] identifiers can be instantly mapped onto UniProt [11] accession numbers, PEDANT [17] protein codes, or EMBL [25] IDs. The PROMPT software facilitates adding new input data types to the mapping procedure by providing an interface for custom input adapters written in *Java*.

### Computable sequence features

In addition to annotation features contained in input files a number of selected characteristics can be calculated directly from protein sequences, mainly using BioJava [24]. These include isoelectric point, the distance of the isoelectric point from neutrality, molecular weight in Daltons, sequence length, grand average hydrophobicity (GRAVY) and the total hydrophobicity of all residues. Additionally the number of alternating hydrophobic/hydrophilic strands is calculated as described in Wong *et al. *[16]. We will be gradually adding additional computable sequence properties driven by our own research needs as well as user requests.

### Statistical analyses

Formally, we are addressing the task of comparing two (protein) datasets in the space of *N *supplied features. PROMPT contains a set of generic engines to analyse and compare nominal as well as numerical attributes. In addition to generating basic descriptive statistics such as mean, standard deviation and median for the distribution of each feature, statistical tests are performed to determine whether the input sets differ significantly with respect to a feature of interest. All statistical tests are encapsulated as *Java *classes and predominantly use the free open source statistical software R [30] or its commercial counterpart S-PLUS [31] as reliable calculation engines. The linkage to R/S is accomplished by PROMPT automatically, assuming R/S is installed in default locations. Alternative and detailed R/S configuration settings can be provided by the user via the GUI config dialog, the XML configuration file, environmental parameters or by or by direct API usage. Although all tests can be chosen manually, PROMPT typically applies the appropriate tests automatically depending on the user's type of input and addressed question. Basically, PROMPT distinguishes four different generic cases: i) comparison of the frequencies of categorical annotations between two sets, ii) enrichment of nominal features in one set with respect to another one, iii) comparison of numeric distributions, and iv) correlation of numeric variables. These four types of analyses are described in more detail below and are also exemplified in Table 2.

#### (i) Feature comparison

The questions handled within this use case are: Are certain categories (e.g. protein functional classes) more frequent in one set or in the other? If yes which ones? And are these differences statistically significant based on respective p-values? PROMPT computes a Chi-Square test for each categorical value that occurs in both sets. Formally, let *A *= {*a*_1_, *a*_2_, ..., *a*_*i *_} *and B *= {*b*_1_, *b*_2_, ..., *b*_*j *_} be sets with *i *and *j *distinct objects and let *V *be the set of nominal categories that can be attributed to the objects. Then each set element can have zero, one or more categorical values assigned. Furthermore let *N*_*a *_and *N*_*b *_be the number of objects of the set *A *and *B *that have at least one category of *V *assigned. Then *frq*_*A *_= *N*_*A *_*|*(*N*_*A *_+*N*_*B *_) and *frq*_*B *_= *N*_*B *_*|*(*N*_*A *_+*N*_*B *_) are the relative frequencies of elements with attributes. Thus only the objects for which annotation data is available are considered.

For each category *v *∈ *V *that is found attributed to objects of A and B a Chi-Square test with the following observation and expectation variables is performed:

##### Observation

*Obs*_*a *_(*v*) = |{*a∈ A*|*v*∈ *attributes*(*a*)}| and *obs*_*b *_(*v*) respectively for the set *B*, i.e. the number of objects in *A *and *B *that have the attribute *v *assigned.

##### Expectation

exp_*A *_(*v*) = (*obs*_*A *_(*v*)+*obs*_*B *_(*v*)))* *frq*_*A *_and exp_*B *_(*v*) = (*obs*_*A *_(*v*)+*obs*_*B *_(*v*)))* *frq*_*B *_, i.e. under the assumption that all variables are independent and identically distributed, exp_*A *_(*v*) and exp_*B *_(*v*) are the number of observations that we would expect if the category *v *is uniformly distributed in *A *and *B*.

The calculation of the Chi-Square test is performed using the Jakarta commons math implementation [32] as the pure JAVA implementation is faster than delegating this simple test.

#### (ii) Feature enrichment

The second method requires the same type of nominal data as in the previous case, but with the additional precondition that one set is a true subset of the other e.g. *A *⊂ *B*. Typical questions that can be answered with this method are: Are up-regulated genes enriched in certain functions? Does the GroEL chaperonin prefer substrates with certain structural folds? Do cancer-associated proteins show non-random enrichment of certain functional families or transcription factor binding sites?

Analogous to the case (i) for each category *v *∈ *V *that is found attributed to objects of A and B, the over- or under representation is calculated and an e-score returns the likelihood that the difference would be found by random. The e-score is calculated as described in Castillo-Davis *et al. *[20] using a hypergeometric distribution with conservative Bonferroni correction.

#### (iii) Comparison of numeric distributions

Are proteins of thermophilic organisms shorter than those of mesophilic organisms [7]? With PROMPT, this question can be answered immediately using its generic method to compare numeric distributions (see our web page, [see Additional file 2]). More generally, the questions that can be answered are: do both sets differ with respect to their means, e.g. are they shifted? Are the distribution functions different? Additionally, for more detailed analyses the distributions can be compared within freely definable intervals, enabling the user to examine whether the protein sets differ within specific ranges of variable values, even if no global differences can be found.

Given two sets of numerical values, PROMPT applies the Mann-Whitney test with the null hypothesis of both distribution functions being equal versus the alternative of the two distribution functions being not equal. The test is sensitive towards differences in the mean, but not towards different variances. Given a continuous distribution function, the two-sample Kolmogorov-Smirnov test checks the null hypothesis that both variables are equally distributed. Both tests can only be applied under the assumption of the variables being independent. They have the advantage that they do not assume the data to follow any specific statistical distribution. By providing the Mann-Whitney and the Kolomogorov-Smirnov test, PROMPT covers both discrete and continuous input data.

For both datasets the key statistical values (such as minimum, maximum, mean, median and standard deviation) as well as histograms with equal binning are calculated. The relative difference of observed values is computed and its significance tested by a Chi-Square test. The Mann-Whitney test is applied to the values of all histogram intervals in order to test whether the distribution functions of the two datasets are identical within each bin.

#### (iv) Correlation of numeric variables

PROMPT provides a generic method to check for correlation between two numeric variables. First, the Pearson correlation coefficient is calculated which is not based on any assumptions about the variables' distributions. Secondly, the Pearson correlation test is performed which expects samples from two independent, bivariate normally distributed distributions. The null hypothesis is that no correlation either negative or positive exists.

### Graphical user interface and scripting capabilities

**Figure 4:**
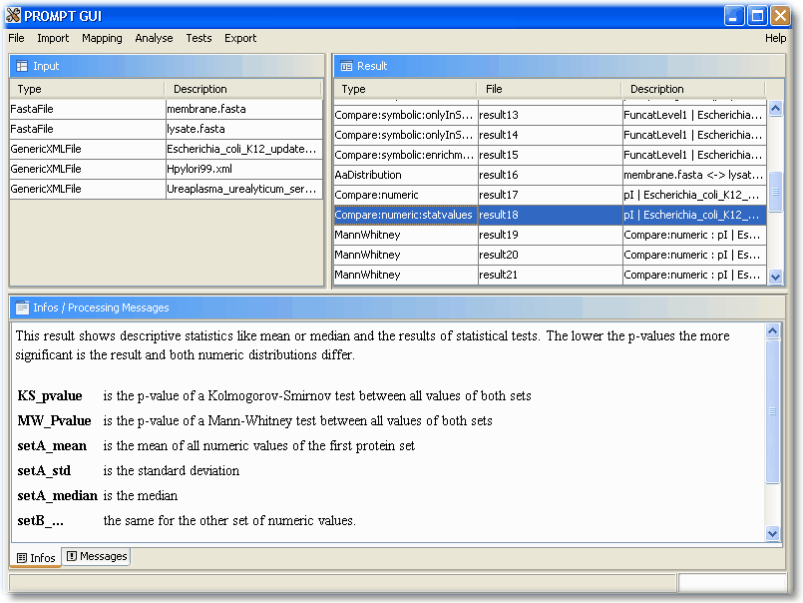
**Graphical User Interface (GUI)**. Shown is a typical workspace session with input data and results. The information panel in the bottom part of the screen provides context sensitive information related to the current user action.

All implemented algorithms can be comfortably run via a stand-alone application with a graphical user interface (GUI), as well as from custom scripts or JAVA programs. The GUI provides a dynamical workspace where input data and results can be managed, analyses performed, statistical tests executed and the results examined, visualized or further processed (Figure 4). All available input adapters, statistical tests and algorithms can be accessed through a menu bar. The menu bar and the GUI itself are fully configurable and extensible by new in-house or third-party modules through XML configuration files or configuration dialogs. The GUI workspace allows confident handling of multiple data sources, analyses, and results, and supports saving and loading any of the input or result objects to/from files. Moreover, the entire workspace can be stored in a compressed form and restored later so that the work on a particular project can be suspended and resumed by the user at any time. The workspace files are portable and can be transferred to other computer systems and shared between different users.

**Figure 5:**
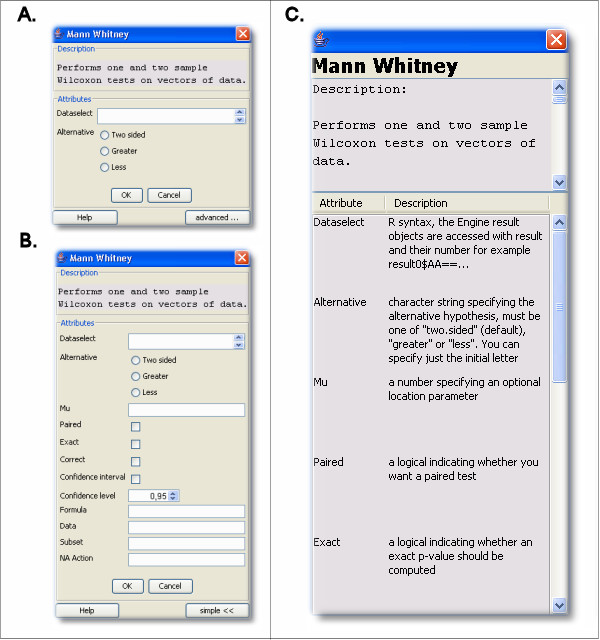
**Screenshots of a typical statistical test dialog**. **A. **The Mann-Whitney test dialog in the simple handling mode with reduced parameters. **B. **The same test in the advanced view with all options allowing full control. **C. **The built-in help with general description of the test and its parameters. The statistical background information was derived from the R documentation.

The PROMPT GUI includes information and message logging panels. The information area displays extensive context-sensitive information about a chosen menu entry or about a selected result entry, providing the user with appropriate hints regarding data integration facilities, available analysis engines, and their results. The message panel shows all logging notes and gives full insight into the analysis progress which is especially useful if longer calculations, such as BLAST similarity searches, are being run. The level of detail and the scope of the logging facility are fully configurable. The data input and retrieval module dialogs guide the user through the data acquisition process and explain various data import features. Likewise, the comparison engines and statistical tests provide context-specific dialogs prompting the user to set or change appropriate parameters. For example, all 27 statistical tests provide individual dialogs (either in simple or advanced mode), tool-tip information, and test specific documentation explaining the meaning of the test and its parameters. These dialogs are rendered automatically from the parameter description of the tests (Figure 5).

Furthermore, a fully searchable and browsable documentation is integrated in the GUI [see Additional file 3]. The GUI provides appropriate actions that match to a chosen result type in a pop-up menu that can be accessed by a right-button mouse click. Via this functionality figures can be generated directly out of the GUI. The GUI checks automatically which of the available plotting classes are applicable to a given data type and allows one to select the desired type of figure.

All of the input, analysis and visualisation functionality is accessible from custom Java programs by utilizing the PROMPT framework classes. Additionally, it is possible to use the whole set of features by writing simple BeanShell [22] scripts as demonstrated in the accompanying examples. BeanShell has the full power of the Java language including access to all Java libraries, and extends it with common scripting capabilities such as loose types, commands, and method closures similar to those in *Perl *and *JavaScript*. In addition to Beanshell scripts, PROMPT can execute conventional Java source code files directly, without the need to compile them. The complete PROMPT framework with all necessary helper classes is provided as one single *jar *library, eliminating the need to conduct extensive Java path configuration.

### Data visualisation and export

**Figure 6:**
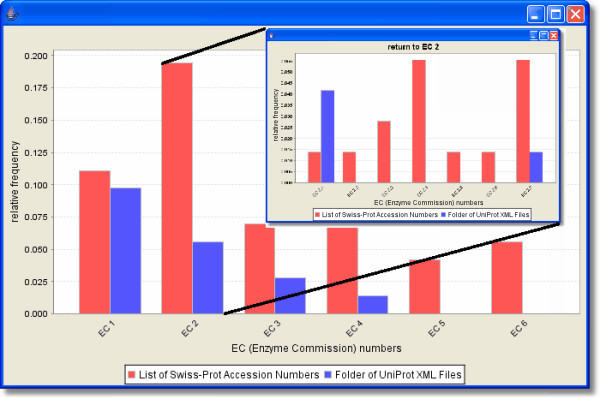
**Example of an interactive browsable figure**. Shown is a comparison of EC numbers found in the annotation of two protein sets. By clicking on the bars the user can zoom in and out the different levels of the Enzyme Nomenclature.

The results of all analyses can be further examined in a graphical spreadsheet view of PROMPT or exported as tab-delimited-, comma-separated- or Microsoft Excel document. Additionally, for the majority of results customized figures can be generated automatically and either saved in the bitmap-oriented portable network graphic (PNG) format or in vector formats such enhanced postscript (EPS) or enhanced windows meta-format (EMF). This allows seamless import of PROMPT results into standard office applications. In some cases, figures produced may be further fine-tuned manually. For example, all underlying data and *R *[30] language commands corresponding to the figures constructed by using *R *as plotting engine can be saved into files. This allows easy customization without the need to run PROMPT analyses again. Another feature is interactive figures (using JFreeChart [33]) as illustrated with the Enzyme-classification viewer of a Swiss-Prot property comparison. By clicking on the enzyme classes it is possible to browse through the different hierarchical levels analysing the functions of interest (Figure 6). The hierarchical category browser is currently restricted to the enzyme classification as available in SwissProt [34]; further categories will follow in subsequent releases of PROMPT. All generic graphical views allow for zooming in or out, inspecting numeric values associated with individual items on the plot, and adjusting the figure appearance in various ways.

## Results

Here, we demonstrate the functionality of PROMPT based on three well documented test cases. Each case study highlights different elementary analysis modes of PROMPT. All used data can be found on the PROMPT home page ([35], [see Additional file 2]), where we additionally provide detailed step-by-step instructions for all cases along with up-to-date information.

**Figure 7:**
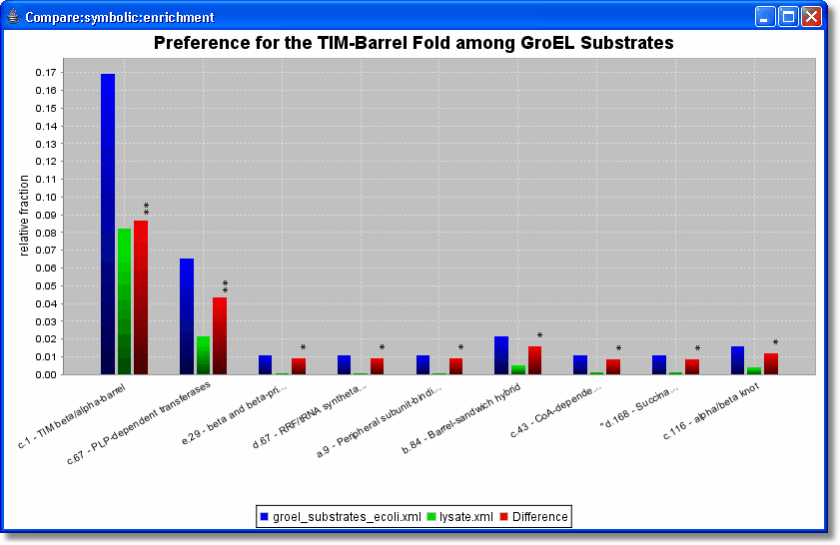
**Example of a categorical comparison analysis**. Frequency of SCOP folds in GroEL substrates compared with the whole E.coli lysate. Only folds that were found at least two times in both sets and that were significantly different at a significance level of 0.05 are shown. The stars on top of the red bars show that the differences are significant with the p-values: < 0.05 *, < 0.01 ** and < 0.001 ***. The figure is a screenshot of an interactive built-in visualisation module provided by PROMPT. All interactive plots allow easy adjustments (changing font sizes, title, axis labels, etc.) and can be saved as graphic files.

In the first case we have reproduced our own previously published analysis of GroEL substrates from *E.coli *[14]. In this work, essentially the entire GroEL-substrate proteome consisting of approximately 250 proteins was identified by a combination of biochemical analyses and quantitative proteomics. What protein features determine substrate specificity of GroEL? To answer this question we imported into PROMPT 20 annotation features for all *E.coli *proteins directly from the PEDANT genome database and compared GroEL substrates with 3202 *E.coli *lysate proteins [36]. The only significant difference reported between these two protein datasets was in terms of their structural folds. Using PROMPT's nominal comparison method we could easily demonstrate that the GroEL substrates are significantly enriched in proteins possessing the TIM-barrel fold (Figure 7). Possible evolutionary implications of this phenomenon are discussed in Kerner *et al*. [14]. Thus, PROMPT allows finding significant enrichments and differences of categorical features between two sets of elements. Furthermore, the generic solution allows an analysis independent of the feature semantic and problem domain.

**Figure 8:**
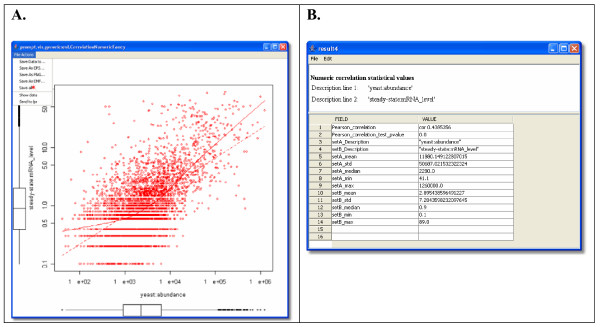
**Results of a correlation analysis**. **A. **Scatter plot of protein abundance against steady-state mRNA expression levels in yeast. The solid and dotted lines show the local polynomial loess fitting curve and the linear regression, respectively. The axes are scaled logarithmically. The box plots visualise the value distribution of each variable. **B. **PROMPT's spread sheet viewer with the Pearson correlation coefficient of 0.44, a highly significant p-value of 0.0 (values below 10^-300 ^are rounded to zero), and further statistical key values. All analysis results can be exported to tab-delimited, comma separated, or Microsoft Excel files.

In the second example we repeat the analysis of protein expression in yeast from Ghaemmaghami *et al. *[37]. This case highlights the ease of using external data with PROMPT, comparing numerical distributions and performing correlation analyses. Absolute protein abundance levels and steady-state mRNA expression levels in *S.cerevisae *were already available as tab-delimited text files associated with the publications by Ghaemmaghami *et al. *[37] and Holstege *et al. *[38], and could be imported easily using PROMPT's tab-delimited input facility. The first question we addressed was whether protein abundance correlates with mRNA expression levels. In addition to calculating the Pearson correlation coefficient PROMPT assesses its statistical significance by performing a correlation test. For visualization of results PROMPT will suggest appropriate options which in this case include a static scatter plot of abundance versus mRNA levels with logarithmic axes and linear- as well as polynomial loess regression lines (Figure 8A). Besides the statistical test results, descriptive key data such as minimum, maximum, mean, median and standard deviation are always returned by PROMPT and can be analysed, sorted and further processed within a comfortable spread sheet viewer as seen in Figure 8B.

**Figure 9:**
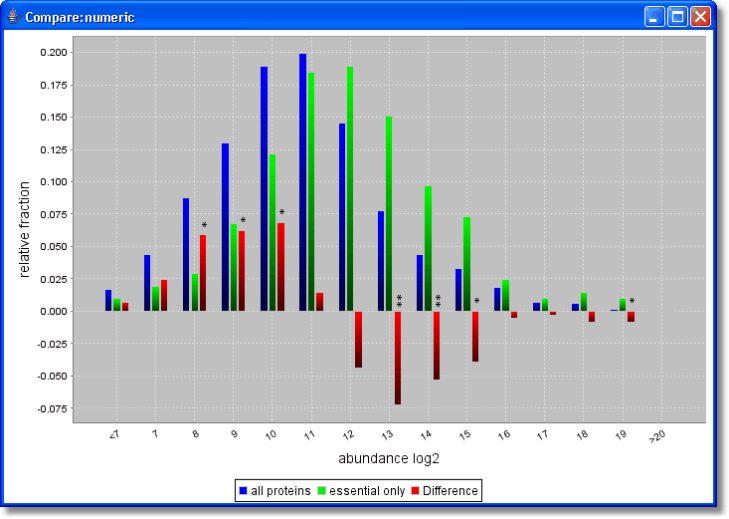
**Comparison of two numeric distributions by PROMPT**. Here normalized abundance distributions of all observed proteins (blue) and essential proteins only (green), as well as the relative difference (red) are shown. These distributions are significantly different (Kolmogorov-Smirnov p-value 6.2 E-12, Mann-Whitney p-value 1.7 E-13). Additionally the stars on top of the red bars show the specific intervals in which the difference is significant. The p-values are indicated by the number of stars: p-value *< 0.05, ** < 0.01 and *** < 0.001.

Another question investigated by Ghaemmaghami *et. al. *[37] was whether essential proteins are more abundant than non-essential proteins. Within a few seconds the results reported by the authors could be reproduced using PROMPT's generic method to compare numerical distributions. Specifically, we compared the abundance distributions of all yeast proteins *vs. *the essential proteins. Applicable statistical tests were automatically performed by PROMPT. First, the value distributions were compared with the Kolmogorov-Smirnov and Mann-Whitney tests based on the complete data set. Secondly, we attempted to identify potential local differences between the two distributions by binning the data and comparing individual bins of both groups separately. This demonstrates that essential proteins are significantly underrepresented within the logarithmic abundance ranges 8 to 11 and significantly overrepresented within the range 13 to 16. The bin intervals can be chosen either automatically or manually guided by a user-friendly graphical dialog box [see Additional file 4]. The resulting comparison of the protein abundance levels of essential proteins *versus *the complete yeast proteome is shown in Figure 9.

In the final example we use PROMPT to automatically retrieve protein sequences by sequence identifiers from public databases and to calculate some of their basic properties such as the isoelectric point. As input we used two lists of GenBank [12] identifiers of membrane and globular proteins of *E.coli*. In this experiment we use only multi-spanning membrane proteins with more than 6 membrane spanning regions predicted by TMHMM 2.0 [39] to avoid any noise from false positive predictions or small membrane-coupled proteins. As seen in the supplementary information [see Additional file 5], longer membrane proteins are less hydrophobic than shorter ones. The observed high correlation between the protein length and its hydrophobicity (expressed as the GRAVY index) of -0.7 is significant with a p-value of 3 E-54. Sequence based properties can also be used in any other generic analysis. For example, the additional figures [see Additional file 6] show a comparison of the automatically derived pI values of membrane and lysate proteins. In addition to the methods based on amino acid sequences, PROMPT provides statistical analyses and comparisons of symbol frequencies of arbitrary alphabets. Thus, in addition to finding over- or under-represented amino acids in a given protein dataset [see Additional file 7], it is also possible to calculate the enrichment/depletion of other symbols such as those taken from the three-state secondary structure alphabet with Helix (H), Strand (E) and Coil (C) as elements.

## Discussion and conclusion

PROMPT is a platform-independent, multi-purpose stand-alone software system for solving a broad spectrum of standard problems in comparative proteomics. It is implemented as a highly-reusable and extensible framework for analysing biological data. With its rich data integration functionality and built-in statistical tests, PROMPT facilitates data mining and hypothesis testing.

PROMPT makes possible incorporation of new algorithms by providing hulls, layers and infrastructure. The availability of both scripting-capability and an intuitive GUI with a context-sensitive help system makes PROMPT equally accessible to both professional bioinformaticians and biologically oriented users. The structure of PROMPT is well adapted for batch processing and automation.

Unlike the multitude of specialized analytical tools, PROMPT has been designed as a versatile general platform for routine analyses and comparisons in the field of molecular bioinformatics. The current version of PROMPT includes a large set of generic comparison methods and statistical tests applicable to any nominal and numeric data as shown in Table 2. User-specific extensions and custom methods can be seamlessly integrated by providing Java classes that implement the interfaces defined in the PROMPT documentation and by adding additional entries to the application's configuration file. Although PROMPT is easily extensible by third-parties, we encourage members of the scientific community to suggest new PROMPT features that may be of particular interest to their research. In the long run we hope to make PROMPT a community resource for comparative proteomics.

## Availability and requirements

**Project name: **PROMPT "Protein Mapping and Comparison Tool"

**Project home page: **

**Operating system(s): **Platform independent

**Programming language: **Java

**Other requirements: **Java 1.5 or higher, R 2.0 (r-project.org) or higher, NCBI Blast 2.1.3 or higher (blastall and formatdb binaries)

**License: **Source code and executables are freely available for academic users from our web site.

**Any restrictions to use by non-academics: **Licence required

## Authors' contributions

TS designed and implemented the software. DF conceived and directed the work. Both authors wrote the article, read and approved the final manuscript.

## Supplementary Material

Additional File 1 Document Type Definition (DTD) of PROMPT's generic XML format Click here for file

Additional File 2 **Screenshot of the PROMPT web page. **Here, we provide the latest news and PROMPT versions along with useful information. Additionally, all case studies shown in this paper including the underlying data are freely available as detailed work-through tutorials. Click here for file

Additional File 3 **Built-in help system. **Comprehensive and intelligent online help with example data and a demonstration workspace allows easy usage of PROMPT without prior knowledge. Click here for file

Additional File 4 **Binning wizard for setting up interval borders**. **A. **First dialog page. The user can either let PROMPT automatically estimate the interval borders, of specify a fixed interval width or the number of intervals. The selected options shown create histogram intervals that have a width of 1, no decimal places, and the range from 6 to 21. **B. **Optional second dialog page. Here the proposed binning can be previewed and altered. Note that we used the special keywords -INF and +INF for negative and positive infinity in the first and last interval to specify that all values less than 7 or higher than 20 fall into these bins. Click here for file

Additional File 5 **Example of a built-in interactive scatter plot. **Protein length of *E.coli *lysate proteins is plotted against their hydrophobicity. The Pearson correlation coefficient is -0.69 with a p-value of 2.8E-54. By pressing and holding the left mouse button it is possible to zoom in the desired area. Clicking on an individual point on the plot leads to numeric values associated with this point being displayed. Click here for file

Additional File 6 **Usage of derived sequence based properties in a generic analysis of PROMPT**. Here the isoelectric point (pI) distributions of the *E.coli *lysate and membrane proteins are compared using the numeric comparison method. PROMPT calculates the pI values automatically if protein sequences are available. Click here for file

Additional File 7 **Screenshots of PROMPT's visualisations of the sequence based symbol analysis methods**. In this example we compared two protein sets with respect of their amino acid composition. The positive and the negative datasets are constituted by the proteins known to crystallize and the proteins whose structure was only resolved by NMR, respectively (Smialowski et al., 2005). **A. **Here the frequencies of each amino acid in both proteins are plotted. For example: a frequency of 5% for threonine in the positive protein dataset means that out of all residues 5% are T's. **B. **Using the same data as in A, here the frequency differences of all sequence elements are shown. For example, the positive value of 0.5% for Y means that this amino acid is about a half percent more frequent is the first dataset. Bars with red color have a significant p-value according to the Mann-Whitney test. **C. **Additionally the frequency distributions of all amino acids can be shown as box plots as exemplified by cysteine here. **D. **Complementary to a box plot depiction PROMPT provides histogram visualizations. Click here for file

## Figures and Tables

**Table 1:** Overview of possible data inputs. Shown are the types of input that can be processed by PROMPT. The Generic XML format can contain any numeric or nominal properties provided by the user.

**Format:**	**Folder with multiple files, each containing one element**	**Individual file with one or more elements**	**List of Identifiers**	**Elements may contain sequences**	**Elements may contain annotation attributes**
FASTA		x		x	
GenBank		x	x	x	
EMBL		x		x	
Swiss-Prot	x	x	x	x	x
UniProt XML	x	x	x	x	x
Generic XML		x		x	x
Tab-delimited		x		x	x
WEKA		x			x

**Table 2:** Summary of PROMPT's generic comparison methods and the corresponding examples presented. The symbol *x *in the data column means corresponding data values for the same protein, whereas a comma simply states that two sets of values are utilized.

**Example**	**Type of data used**	**PROMPT method**^a^**:**	**Applied statistical methods **^d^
Fold comparison of GroEL substrates with the whole proteome	**{ Nominal }, { Nominal }**	Categorical feature comparison ^b^	• Chi-Square test
Fold enrichment of GroEL substrates	**{ Nominal }, subset of { Nominal }**	Categorical feature enrichment ^c^	• Sampling from hypergeometric distribution with correction
Abundance distribution of essential vs. all proteins	**{ Numeric }, { Numeric }**	Numeric distribution comparison	• Mann-Whitney (MW) and Kolmogorov-Smirnov (KS) of the whole distribution • MW and Chi-Square test of each bin separately
Protein abundance vs. mRNA expression	**{ Numeric × Numeric }**	Numeric feature correlation	• Pearson correlation coefficient and • Pearson correlation test

^a^ Extensive description of each method can be found in the context sensitive help integrated in the PROMPT GUI, or in the manual supplied with PROMPT.

^b ^Both groups with categorical data can be independent from each other.

^c ^One group must be drawn from the other group.

^d ^As described in the *Methods *section
