# Supplementary material for: PROMPT: a protein mapping and comparison tool
Source: BMC Bioinformatics. 2006 Jul 4;7:331. doi: 10.1186/1471-2105-7-331 (PMC1569443; doi:10.1186/1471-2105-7-331)
Supplement: Additional File 1 — Document Type Definition (DTD) of PROMPT's generic XML format [file 1471-2105-7-331-S1.pdf]

```
<?xml version="1.0" encoding="UTF-8"?>
<!--DTD for the generic XML format-->
<!ELEMENT dataset (property+)>
<!ELEMENT property (input+)>
<!ELEMENT input EMPTY>
<!ATTLIST dataset
      label CDATA #REQUIRED
      version CDATA #IMPLIED
>
<!ATTLIST property
      id CDATA #REQUIRED
      type (symbolic | numeric) #REQUIRED
>
<!ATTLIST input
      id CDATA #IMPLIED
      value CDATA #REQUIRED
>
```
