# Supplementary figures and images for: PROMPT: a protein mapping and comparison tool
Source: BMC Bioinformatics. 2006 Jul 4;7:331. doi: 10.1186/1471-2105-7-331 (PMC1569443; doi:10.1186/1471-2105-7-331)

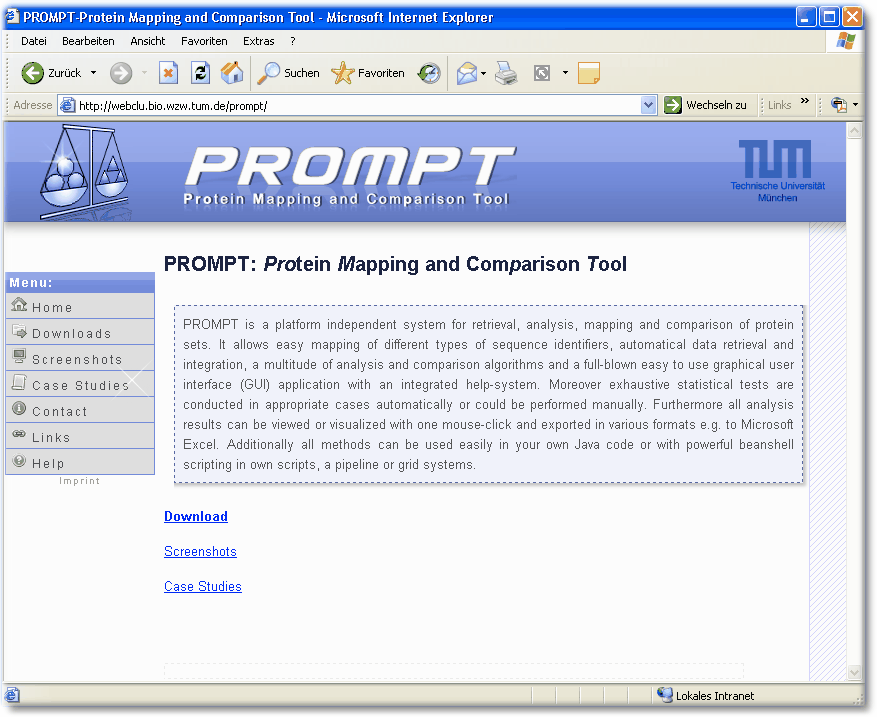

Supplement: Additional File 2 — Screenshot of the PROMPT web page. Here, we provide the latest news and PROMPT versions along with useful information. Additionally, all case studies shown in this paper including the underlying data are freely available as detailed work-through tutorials. [file 1471-2105-7-331-S2.png]

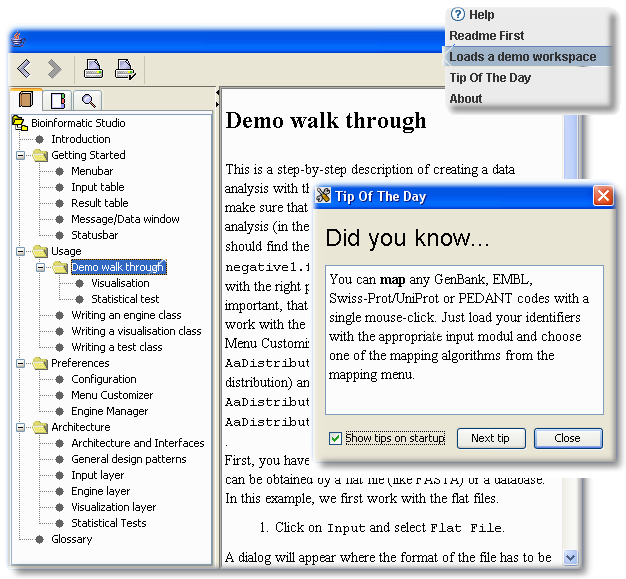

Supplement: Additional File 3 — Built-in help system. Comprehensive and intelligent online help with example data and a demonstration workspace allows easy usage of PROMPT without prior knowledge. [file 1471-2105-7-331-S3.png]

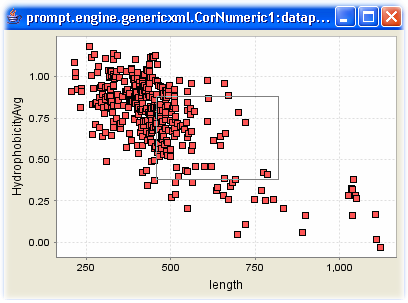

Supplement: Additional File 5 — Example of a built-in interactive scatter plot. Protein length of E.coli lysate proteins is plotted against their hydrophobicity. The Pearson correlation coefficient is -0.69 with a p-value of 2.8E-54. By pressing and holding the left mouse button it is possible to zoom in the desired area. Clicking on an individual point on the plot leads to numeric values associated with this point being displayed. [file 1471-2105-7-331-S5.png]

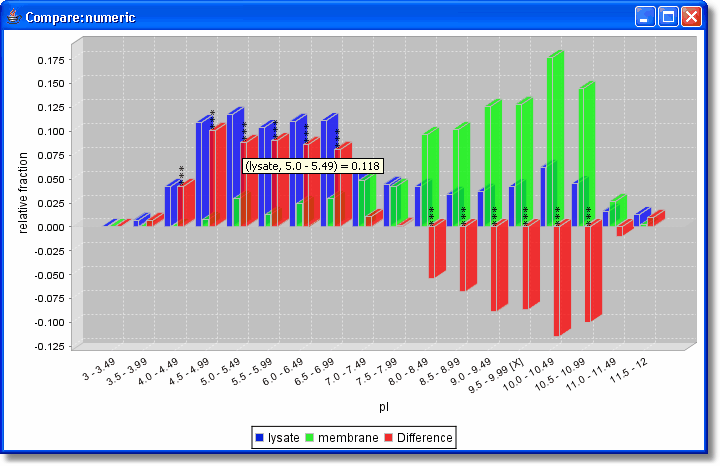

Supplement: Additional File 6 — Usage of derived sequence based properties in a generic analysis of PROMPT. Here the isoelectric point (pI) distributions of the E.coli lysate and membrane proteins are compared using the numeric comparison method. PROMPT calculates the pI values automatically if protein sequences are available. [file 1471-2105-7-331-S6.png]

**A.**

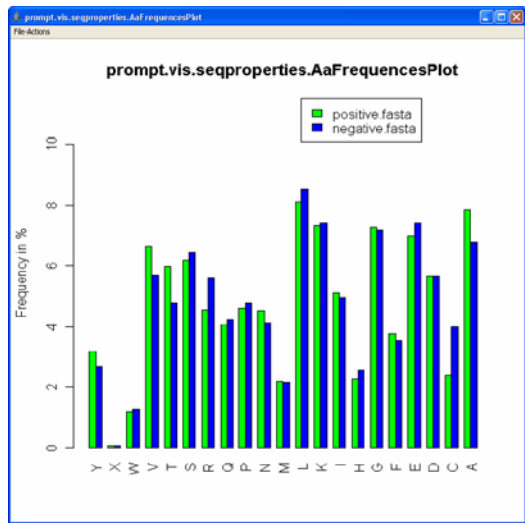

**B.**

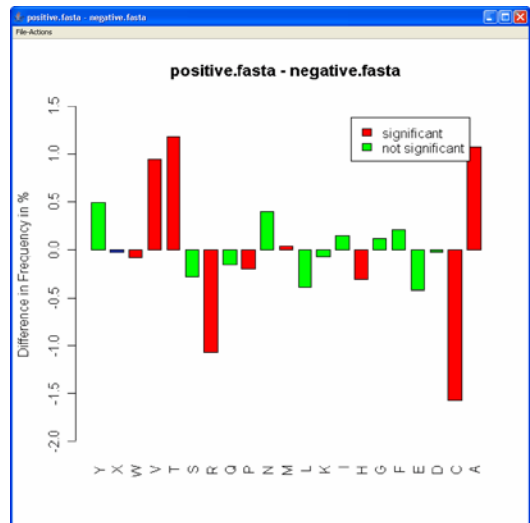

**C.**

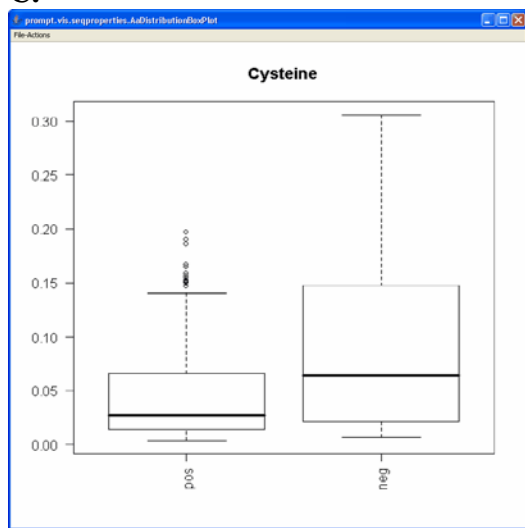

**D.**

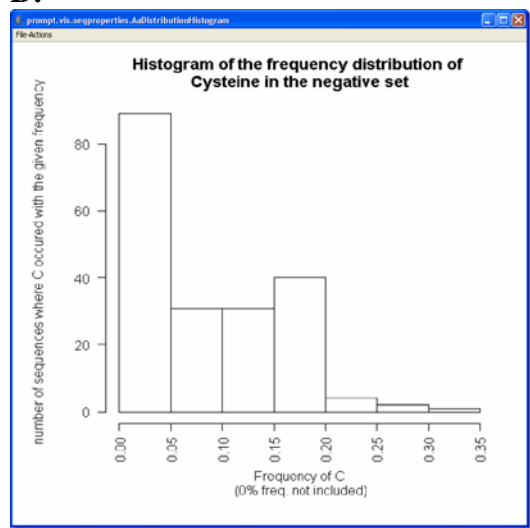

Supplement: Additional File 7 — Screenshots of PROMPT's visualisations of the sequence based symbol analysis methods. In this example we compared two protein sets with respect of their amino acid composition. The positive and the negative datasets are constituted by the proteins known to crystallize and the proteins whose structure was only resolved by NMR, respectively (Smialowski et al., 2005). A. Here the frequencies of each amino acid in both proteins are plotted. For example: a frequency of 5% for threonine in the positive protein dataset means that out of all residues 5% are T's. B. Using the same data as in A, here the frequency differences of all sequence elements are shown. For example, the positive value of 0.5% for Y means that this amino acid is about a half percent more frequent is the first dataset. Bars with red color have a significant p-value according to the Mann-Whitney test. C. Additionally the frequency distributions of all amino acids can be shown as box plots as exemplified by cysteine here. D. Complementary to a box plot depiction PROMPT provides histogram visualizations. [file 1471-2105-7-331-S7.pdf]
