# Supplementary material for: PROMPT: a protein mapping and comparison tool
Source: BMC Bioinformatics. 2006 Jul 4;7:331. doi: 10.1186/1471-2105-7-331 (PMC1569443; doi:10.1186/1471-2105-7-331)
Supplement: Additional File 4 — Binning wizard for setting up interval borders. A. First dialog page. The user can either let PROMPT automatically estimate the interval borders, of specify a fixed interval width or the number of intervals. The selected options shown create histogram intervals that have a width of 1, no decimal places, and the range from 6 to 21. B. Optional second dialog page. Here the proposed binning can be previewed and altered. Note that we used the special keywords -INF and +INF for negative and positive infinity in the first and last interval to specify that all values less than 7 or higher than 20 fall into these bins. [file 1471-2105-7-331-S4.pdf]

A.

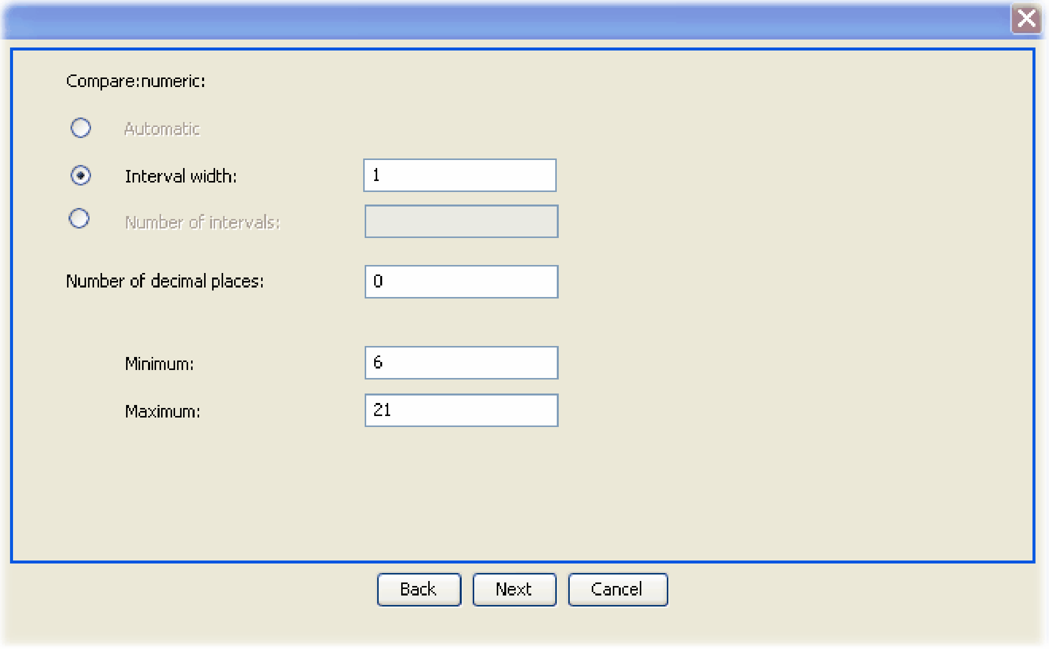
Compare:numeric:
Automatic
Interval width:
1
Number of intervals:
Number of decimal places:
0
Minimum:
6
Maximum:
21
Back
Next
Cancel

B.

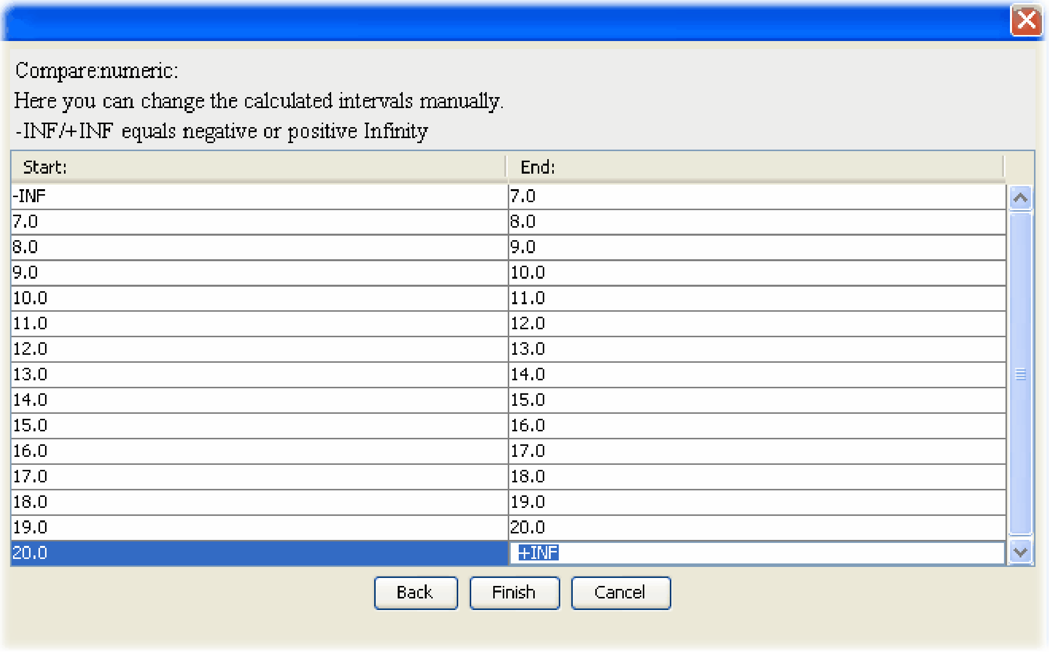
Compare:numeric:
Here you can change the calculated intervals manually.
-INF/+INF equals negative or positive Infinity
Start:
End:
-INF
7.0
7.0
8.0
8.0
9.0
9.0
10.0
10.0
11.0
11.0
12.0
12.0
13.0
13.0
14.0
14.0
15.0
15.0
16.0
16.0
17.0
17.0
18.0
18.0
19.0
19.0
20.0
20.0
+INF
Back
Finish
Cancel
